# Clinical analysis of 44 cases of atypical polypoid adenomyoma of the uterus

**DOI:** 10.1186/s12905-022-01643-y

**Published:** 2022-03-04

**Authors:** Xin Wang, Yinshu Guo

**Affiliations:** Department of Gynecology Minimally Invasive Center, Beijing Obstetrics and Gynecology Hospital, Capital Medical University, Beijing Maternal and Child Health Care Hospital, 17 Qihelou Street, Dongcheng District, 100006 Beijing, China

**Keywords:** Atypical polypoid adenomyoma, Hysteroscopy, Therapy, Follow-up

## Abstract

**Background:**

Atypical polypoid adenomyoma (APA) is a rare intrauterine polypoid lesion that occurs predominantly in premenopausal women. Although APA was previously considered a benign lesion and treated conservatively, an increasing number of cases show that APA has a high rate of recurrence or residual disease and that it precedes the development of carcinoma. The clinical management of APA remains to be established. The aim of this study was to analyse the clinicopathological features of APA and discuss its diagnosis and prognosis.

**Methods:**

Forty-four patients with APA were admitted to Beijing Obstetrics and Gynecology Hospital from 2005 to 2019, and their clinical and histopathologic features were evaluated. B-ultrasound was performed, and all the patients (n = 44) underwent hysteroscopy. Endometrium excision was performed by means of the four-step diagnosis and treatment method. Hysteroscopic transcervical resection (TCR) was performed in 5 patients with APA-H and 11 with APA-L. Except for one patient who underwent transcervical endometrial resection, all the patients underwent hysterectomy and salpingectomy or salpingo-oophorectomy. Data from a median follow-up of 42 months (ranging from 3 to 174 months) were available for these patients.

**Results:**

Pathological diagnosis were made according to the degree of abnormality of the APA surface glands, resulting in APA-L in 36 patients and APA-H in 8 patients. Among these patients, 28 (25 APA-L and 3 APA-H) were treated conservatively. The effect of the four-step diagnosis and treatment method as an APA therapy was excellent. During the follow-up, no evidence of recurrence was found.

**Conclusions:**

For patients with intracavitary lesions > 1 cm, the hysteroscopic four-step diagnosis and treatment method and pathological diagnosis are the basis of clinical treatment. More than 30% of APA surface glands have complex structures characterized by branching and budding or other high-risk factors, such as endometrial hyperplasia, which are indications for hysterectomy. For patients who desire to become pregnant or to preserve the uterus, hysteroscopy with complete excision of the lesions should be the preferred treatment method. The patients should be treated and followed up closely with regular hysteroscopy and endometrial biopsy.

## Introduction

Many common gynaecologic conditions, such as endometriosis or endometrial polyps, are associated with infertility [1, 2]. Atypical polypoid adenomyoma (APA) is a rare intrauterine space-occupying lesion composed of atypical endometrial glands surrounded by smooth muscle tissue bundles [3]. The term APA was first proposed by Mazur in 1981. Five types of polypoid lesions, characterized by atypical glands with squamous metaplasia and smooth muscle intercellular stroma, in premenopausal women have been described [4].

APA is a rare and benign uterine tumour, and it is difficult to evaluate its incidence [5]. Most APA patients are reproductive-aged women, but some studies have reported APAs in postmenopausal women [6]. Abnormal uterine bleeding, anaemia and infertility are the main clinical symptoms of APA [7]. Preoperative examinations, including B-ultrasound, CT and MRI, can be used to preliminarily evaluate the condition of the uterus, and hysteroscopy technology allows comprehensive assessments of endometrial lesions with the naked eye. However, precisely discriminating APA from endometrial polyps with the naked eye is not possible. Consequently, histological examination for the pathological diagnosis of APA is currently the diagnostic standard [8, 9].

The size of the tumour diameter varies from 0.1 to 6 cm, with 12 cm being the largest recorded tumour. Usually, the lesion arises in the lower uterine segments, sometimes in the uterine fundus or endocervical canal, but it has also been reported to involve the oviduct in monkeys. Histologically, APA consists of biphasic proliferation of atypical endometrial glands with squamous morular differentiation and abundant stroma with smooth muscle and fibrous tissue. The myofibromatous stromal tissue derives from the myofibromatous metaplasia of endometrial stromal cells. Some evidence indicates that prolonged oestrogenic stimulation may be significant. In addition, Longacre et al. proposed that if the lesions of APA exhibit markedly complex glands and severe architectural complexity, these PAPs should be designated APA of low malignant potential (APA-LMP), emphasizing the potential risk related to malignant potential. Findings from previous immunohistochemical studies have revealed that the stromal components of PAP are positive for a-smooth muscle actin (a-SMA), oestrogen receptor (ER), progesterone receptor (PR), P53, Ki-67, CD34 and desmin but negative or weakly positive for CD10 and h-caldesmon.

However, there is currently no gold standard for the clinical treatment of APA patients. Medication and surgery are the main treatment modalities for patients with APA [10], and fertility preservation treatment is performed in women of childbearing age who wish to become pregnant. Patients treated with conservative therapy have a high rate of relapse and endometrial adenocarcinoma [11–13]. Once a patient is diagnosed with APA, a long-term follow-up becomes necessary. The current study retrospectively investigated the clinical characteristics, treatment and prognosis of APA to provide suggestions for the diagnosis and treatment of APA.

## Methods and materials

### Study population

We collected clinical information and pathology specimens from 44 patients with APA who had been diagnosed and treated at the gynaecological minimally invasive centre of Beijing Obstetrics and Gynaecology Hospital between 2005 and 2019. The clinical manifestations in this group were as follows. (1) A total of 35 patients had abnormal uterine bleeding, with 26 having menstrual changes and 9 having postmenopausal bleeding. Among the 26 patients with menstrual changes, there were 10 cases of menstrual disorder, 8 of prolonged menstruation, 4 of increased menstrual volume and 4 of intermenstrual bleeding. (2) Among the 44 patients, there were 8 with no obvious clinical symptoms but an abnormal echo or heterogeneous thickening of the endometrium based on the B-ultrasound examination. (3) Among the 44 patients, there were 7 cases of infertility. (4) Additionally, there were 2 cases of dysmenorrhoea, 2 of vaginal fluid and 2 of hypogastralgia. There were no obvious differences among the gynaecological examinations. B-ultrasound examinations showed an abnormal echo in the uterine cavity or cervical canal in 25 patients, endometrial thickening or an abnormal intrauterine echo in 17 patients, and no obvious abnormality in 2 patients. Twenty-one patients had blood flow signals such as star-like blood flow or ri0.32–0.47 on B-ultrasound. This patient group did have clinical complications. There were 6 patients with APA combined with hysteroscopic endometrial polyp or cervical polypectomy, 8 with APA combined with hysteromyoma, 1 with APA combined with benign ovarian tumour, 10 with APA combined with abnormal uterine bleeding-ovulation disorder, 10 with APA combined with hypertension, 4 with APA combined with diabetes, 2 with APA combined with hyperthyroidism, and 1 with APA who had taken toremifene citrate for 5 years after breast cancer surgery (Table 1).

**Table 1:** Patient information

No	Age	BMI	CA125	Menopausal status	AUB	Infertility	Ultrasound finding	Haemoglobin
1	28	31.94	36.1	Premenopausal	No	No	Endometrial thickening	134
2	33	28.24	30.5	Premenopausal	No	No	Endometrial thickening	144
3	35	31.15	68.5	Premenopausal	Yes	No	Normal	122
4	28	31.31	60.7	Premenopausal	Yes	No	Endometrial thickening	151
5	40	34.91	17.2	Premenopausal	Yes	Yes	Abnormal	111
6	30	31.31	41.8	Premenopausal	Yes	No	Abnormal	142
7	36	24.05	64.2	Premenopausal	Yes	Yes	Endometrial thickening	115
8	39	27.42	54.8	Premenopausal	Yes	No	Endometrial thickening	116
9	47	25.84	9.3	Premenopausal	Yes	No	Endometrial thickening	134
10	53	23.48	37.3	Premenopausal	No	No	abnormal	113
11	33	22.32	5.6	Premenopausal	No	Yes	Endometrial thickening	122
12	39	23.62	18.9	Premenopausal	Yes	No	abnormal	140
13	48	29.43	39.8	Premenopausal	Yes	No	Endometrial thickening	145
14	58	34.11	41.3	Premenopausal	Yes	Yes	Endometrial thickening	133
15	73	22.68	6.9	Postmenopausal	Yes	No	Endometrial thickening	127
16	35	22.11	35.3	Premenopausal	No	Yes	Endometrial thickening	151
17	36	30.58	53.4	Premenopausal	yes	Yes	Abnormal	141
18	41	25.78	40.5	Premenopausal	Yes	No	Endometrial thickening	113
19	47	22.28	36.4	Premenopausal	Yes	No	Endometrial thickening	128
20	54	31.98	18.7	Premenopausal	Yes	No	Endometrial thickening	96
21	38	32.98	16.7	Premenopausal	No	No	Endometrial thickening	134
22	43	33.09	45.7	Premenopausal	Yes	No	Abnormal	112
23	47	25.51	36.7	Premenopausal	Yes	No	Abnormal	139
24	49	22.1	55.6	Premenopausal	Yes	No	Abnormal	124
25	57	32.24	11.9	Premenopausal	No	No	Endometrial thickening	141
26	73	30.63	34	Postmenopausal	Yes	No	Abnormal	132
27	74	29.75	12.1	Postmenopausal	Yes	No	Endometrial thickening	141
28	39	29.13	26.7	Premenopausal	Yes	Yes	Endometrial thickening	106
29	62	30.17	38.4	Postmenopausal	No	No	Endometrial thickening	147
30	70	24.1	33.7	Postmenopausal	No	No	Endometrial thickening	118
31	59	30.66	19.2	Premenopausal	Yes	Yes	Endometrial thickening	121
32	61	24.88	69.7	Premenopausal	Yes	No	Abnormal	111
33	69	24.8	68	Postmenopausal	Yes	No	Endometrial thickening	121
34	61	32.34	56.6	Premenopausal	No	Yes	Abnormal	90
35	63	33.57	4.3	Postmenopausal	Yes	No	Abnormal	121
36	66	25.97	12.2	Postmenopausal	No	No	Abnormal	140
37	73	22.63	63.6	Postmenopausal	Yes	No	Endometrial thickening	108
38	42	29.44	8	Premenopausal	Yes	No	Abnormal	113
39	47	23.98	14.4	Premenopausal	Yes	No	Abnormal	127
40	53	25.16	5.5	Premenopausal	Yes	Yes	Abnormal	132
41	61	23.29	12.6	Premenopausal	Yes	Yes	Endometrial thickening	142
42	61	26.92	28.2	Postmenopausal	Yes	No	Normal	167
43	73	25.93	26.9	Postmenopausal	No	No	Endometrial thickening	132
44	73	25.5	26.7	Postmenopausal	Yes	No	Abnormal	119

### Four-step diagnosis and treatment

All the patients underwent hysteroscopy and resection of uterine cavity-occupying lesions. A four-step diagnosis and treatment strategy was used for endometrium excision as follows: (1) complete excision of occupying lesions from the root; (2) resection of endometrial tissue around the root (ranging from 0.2 to 0.5 cm); (3) removal of 0.3 cm of myometrial tissue below the root; and (4) hysteroscopy and multipoint biopsy of the remaining endometrium. All the resected tissues were submitted for pathological diagnosis, which is the basis of clinical treatment.

## Results

### Hysteroscopic findings

**Fig. 1:**
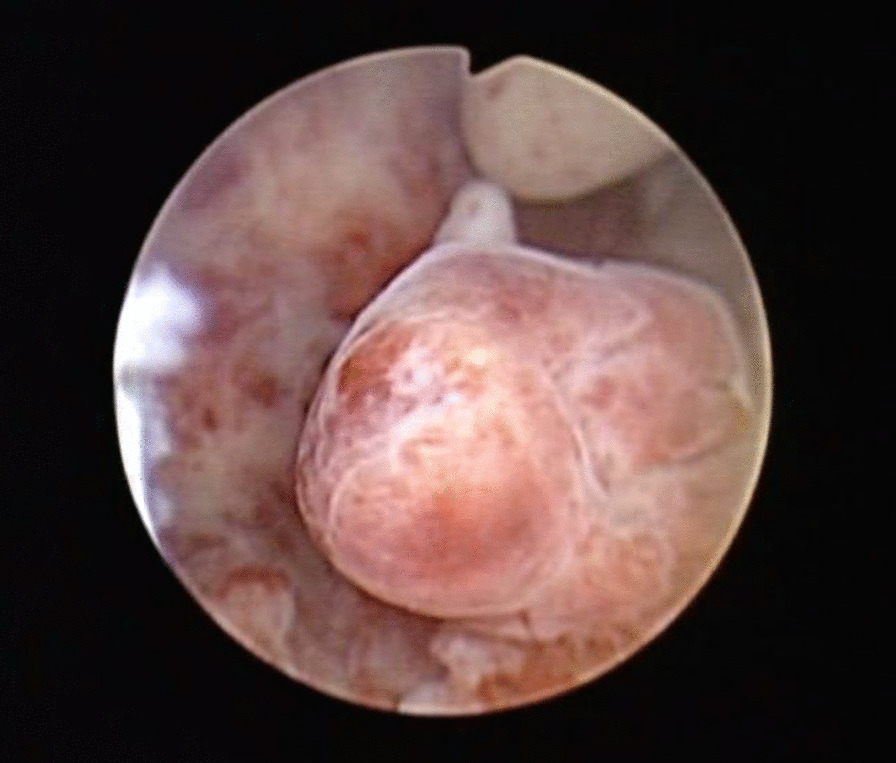
Atypical polypoid adenomyoma seen on hysteroscopy (lesions located in the posterior wall of the uterine cavity, diameter 2.0 cm, irregular shape)

All 44 patients underwent hysteroscopy. A single lesion was found in 40 patients (90.9%), and the mean size was 2.83 ± 0.73 cm (ranging from 0.5 to 6 cm). The specific distribution of the lesions was as follows: 10 cases in the uterine cavity bottom, 8 in the anterior wall, 7 in the left uterine angle, 5 in the right uterine angle, 5 in the posterior wall, 2 in the right anterior wall, 1 in the right wall, 1 in the right anterior wall near the cervical intraoral cavity, and 1 in the left wall. The surfaces of 8 lesions had rich blood vessels, and 2 lesions had atypical vessels, which is a typical hysteroscopic morphology (Fig. 1). In addition, 4 patients had multiple lesions.

### Pathological diagnosis

**Fig. 2:**
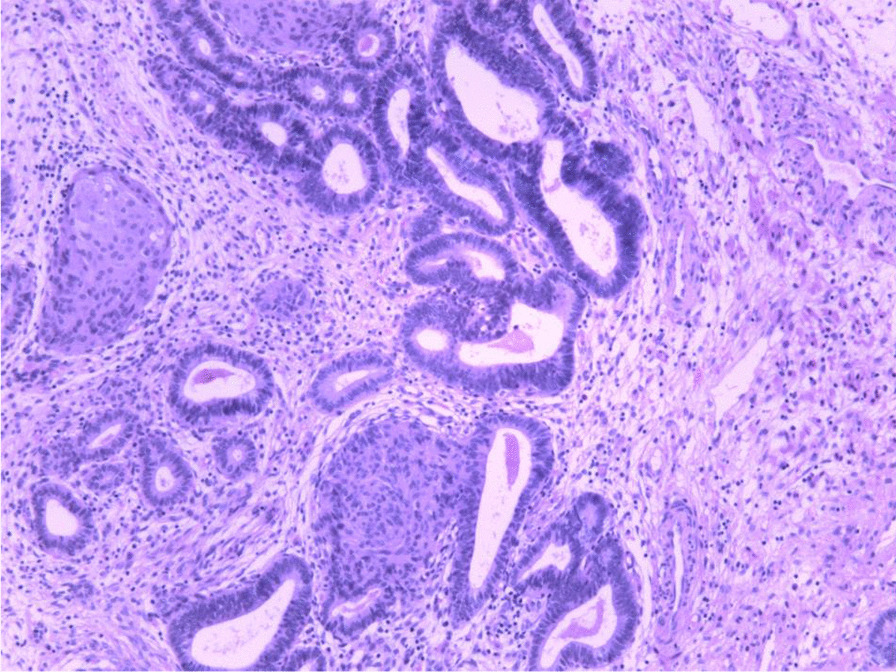
Representative microscopic appearance of atypical polypoid adenomyoma (H&E stain)

APA was diagnosed pathologically after hysteroscopic resection of the lesion (Fig. 2). According to the degree of abnormality of the APA surface glands, APA was classified as APA-L or APA-H [11]. More than 30% of the APA surface glands had complex structures characterized by branching and budding (Fig. 2), in which dense glands coexisted with branched glandular duct structures. There were 36 cases of APA-L and 8 cases of APA-H. Hysteroscopic biopsy of the endometrium in other parts of the uterine cavity revealed mild or moderate atypical hyperplasia in 7 patients.

### APA association with medication

Twenty-eight patients aged 27 to 42 years with the desire for uterine preservation, including 15 patients who attempted to conceive, were treated conservatively. Three patients with APA-H and two patients with APA-L combined with mild atypical hyperplasia of the endometrium were treated with medroxyprogesterone acetate (500 mg/day). After 3–6 months of drug treatment, hysteroscopy and multipoint endometrial biopsy were performed. When the endometrial pathological diagnosis was associated with the effect of progesterone treatment, such as the development of partial glands in the endometrium during the secretory phase or atrophy during the proliferative phase, the high-performance progestogen administration was stopped. Patients began taking medroxyprogesterone on the 14th day of menstruation (20 mg/day) for 12 days. After 3 months of medication, hysteroscopy and endometrial biopsy were performed, and no abnormal changes in the endometrium were found.

**Fig. 3:**
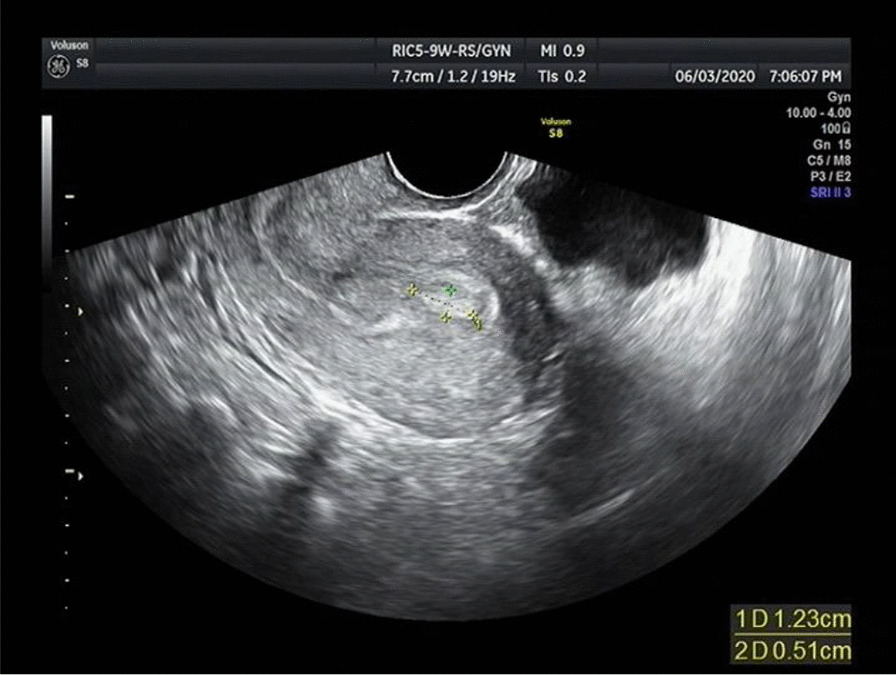
Atypical polypoid adenomyoma on B-ultrasound (endometrium 1.0 cm, intrauterine hyperechoic mass 1.7*1.2 cm)

The drug treatment for 23 patients with APA-L was as follows: 8 were treated with high-performance progestogen, 6 were given medroxyprogesterone or progesterone in the second half of the menstrual cycle, 4 were on short-acting oral contraceptives, and 5 were followed up regularly without medication. Hysteroscopy and endometrial biopsy were performed every 3 to 6 months to evaluate the histological changes in the endometrium. If the endometrium was normal with two consecutive endometrial biopsies, then regular hysteroscopy and endometrial biopsy were no longer needed. In addition, menstruation and transvaginal ultrasound were performed to look for abnormal growth of the endometrium showing the characteristic B-ultrasound appearance of APA (Fig. 3).

### APA association with surgery

In total, 16 patients received surgery. One patient underwent transcervical endometrial resection because hysteroscopic biopsy showed complex endometrial hyperplasia. Fifteen patients with total hysterectomy and salpingectomy or salpingo-oophorectomy were 44 to 74 years old with no desire for uterus preservation and conception. Among those who underwent endometrial biopsy, 5 patients had APA-H, 5 patients had mild or moderate atypical hyperplasia of the endometrium, and 4 patients had uterine leiomyoma or abnormal uterine bleeding. After total hysterectomy, endometrial pathological diagnosis indicated 4 patients with mild atypical hyperplasia, 1 with mild or moderate atypical hyperplasia, and 11 with benign endometrial tissues.

### APA association with follow-up

The patients were followed up for 6–174 (62.5 ± 45.3) months, and there was no recurrence among the patients who received conservative treatment. (1) Among 15 patients who desired to give birth, 3 with ovulation disorders gave up on attempts to conceive after assisted reproduction technology failed. However, 12 patients became pregnant after hysteroscopic resection of their uterine cavity-occupying lesions, including 7 with spontaneous conception and 5 with increased odds of a successful pregnancy by ovulation induction or assisted reproductive technology. (2) Seven patients with abnormal uterine bleeding and ovulatory dysfunction experienced abnormal menstruation. Among them, 3 received progestin treatment in the second half of the menstrual cycle, 3 underwent an insertion of the levonorgestrel-releasing intrauterine system, and 1 who was followed up for 52 months after hysteroscopic conservative surgery had post-hysteroscopy and endometrial biopsy pathology results that revealed mild atypical hyperplasia of the endometrium, leading to total laparoscopic hysterectomy and salpingectomy.

## Discussion

The incidence of APA is low, and the cause of the disease is currently unclear. It has been reported that the age of onset of APA is 18 to 81 years [11] and that APA occurs mostly in premenopausal women [14]. The patients in the current study were 27 to 74 years old (mean age, 45.1 ± 13.7 years) 32 (72.7%) of these patients were premenopausal, and 12 (27.3%) were postmenopausal. The most common clinical manifestation was abnormal uterine bleeding (35/44). The second most common characteristic of the patients in our group was the B-ultrasound finding of no obvious symptoms in terms of an intrauterine echo (8/44). Other clinical complications, including infertility (7/44), AUB-O (10/44) and diabetes (4/44), were found in this group. Notably, one patient received toremifene citrate for 5 years after breast cancer surgery. Seven patients had mild or moderate atypical hyperplasia, considering that the occurrence of APA is related to continuous oestrogen stimulation.

Patients with APA do not have typical specific clinical manifestations. The most common symptom is abnormal uterine bleeding [15]. Auxiliary tools such as gynaecological ultrasonography for pelvic examination can be performed in sexually active women; otherwise, transrectal ultrasonography is considered [16]. Ultrasound techniques are now routine methods for first-level screening in patients with suspected endouterine disease because of their low cost, reproducibility and absence of complications [17]. Transvaginal ultrasound can indicate heterogeneous endometrial thickening, abnormal intrauterine echoes, and blood flow changes without specificity. Therefore, it is necessary to differentiate APA from endometrial polyps, endometrial cancer (EC), adenomyosis, uterine adenofibroma and malignant mixed Mullerian tumours. APA can be combined with precancerous endometrial lesions and EC [18]. Additionally, hysteroscopy should be performed for patients with clear indications, such as abnormal uterine bleeding, abnormal intrauterine echo and infertility. 3D sonohysterography has been reported to be a good method of screening for hysteroscopic confirmation, especially in patients with suspected polyps, myoma, mucus accumulation and Mullerian anomalies [17]. During hysteroscopy, endometrial thickness, texture, vascular morphology, intrauterine lesions, size, location, texture and surface vascular characteristics of the lesions should be carefully evaluated. The reliability of hysteroscopy in diagnosing focal intrauterine lesions even in precancerous cases has been shown in many previous studies. Data and statistical analyses in our study showed that the combination of transvaginal ultrasound and hysteroscopy plays an important role in the identification of APA lesions.

Among the patients in this group, 40 had single lesions. The diameters of the lesions were 0.5 to 6 cm, with an average diameter of 2.83 ± 0.73 cm, which was consistent with the literature [19]. APA does not have a unique appearance under hysteroscopy, and it is often confused with endometrial polyps or submucosal fibroids. However, the diameters of APAs are larger than 1 cm in most cases, with the lesion surface consisting of abundant and thick blood vessels. Therefore, during surgery, uterine space-occupying lesions with diameters greater than 1 cm should be completely resected according to the four-step diagnosis and treatment method used in this study. Additionally, corresponding biopsies of the endometrium and superficial muscular layer at the base and its surrounding area should be performed. It is indispensable to follow-up the pathological diagnosis to decrease the possibility of a misdiagnosis.

Wong et al. found that progesterone may have a protective effect in APA patients during pregnancy [20]. Chen et al. demonstrated that APA patients who desired to give birth and were treated with progestin had no recurrence after undergoing hysteroscopic resection of the lesion [21]. Zhang et al. [22] revealed that the four-step diagnosis and treatment method is the most effective treatment for APA patients, as it completely reduces the recurrence rate. However, other research has indicated that the recurrence rate of APA in patients ranges from 28.9% to 35.1% [14, 18, 23], as deeper invasion into the uterine muscle is easily induced. A multicentre study revealed that the malignant transformation rate of APA is up to 0.8%, which is much higher than that of endometrial polyps [24].

Therefore, APA treatments can be individualized according to age, fertility requirements and postoperative pathological diagnosis. Total hysterectomy is recommended for menopausal or perimenopausal patients with APA-H. Additionally, in determining precise treatments for patients with APA-L, physicians need to consider the patient’s age and desire to become pregnant or to preserve the uterus. High-efficiency progesterone therapy is recommended for patients of childbearing age with APA-H. Moreover, patients of childbearing age with APA-L should undergo regular follow-up. In this study, patients with APA-H or APA-L combined with atypical hyperplasia of the endometrium were treated with high-efficiency progesterone. Patients with APA-L were treated with progestin in the second half of the menstrual cycle and with short-acting oral contraceptives and then followed up regularly without medication so that there would be no recurrence in this group. Regular postoperative follow-up measures were performed for APA. Patients with APA-H or atypical hyperplasia of the endometrium tend to undergo uterine preservation or give birth, so this group should receive regular and close follow-up. Hysteroscopy and endometrial biopsy are the basis of treatment schemes and decrease the misdiagnosis rate of endometrial diseases. A recent metanalysis indicated that the best treatment for APA is hysteroscopy. Medications, in particular progestogens, are not the first-line treatment but could prevent APA recurrence [5].

Forty-four patients were confirmed to have no recurrence by regular hysteroscopy and endometrial biopsy during follow-up. There are many reasons for this outcome. First, complete resection of the lesion according to the principles of the four-step diagnosis and treatment method is the main treatment for APA patients, as this reduces the rate of misdiagnosis and provides an effective foundation for clinical treatments. Second, follow-up is of great significance for patients with conservative treatments. Transvaginal ultrasound, hysteroscopy and endometrial biopsy were combined during follow-up to avoid false negatives and improve the accuracy of endometrial biopsy. One patient in this group was found to have mild atypical hyperplasia of the endometrium by hysteroscopy and endometrial biopsy during follow-up. Surgery was performed in this patient to avoid malignant transformation of the endometrium. Finally, continuous stimulation with oestrogen and a lack of progesterone are the main pathological mechanisms of APA. Therefore, a levonorgestrel-releasing intrauterine device is the first choice for the treatment of APA patients with abnormal uterine bleeding and ovulatory dysfunction. In addition, these patients need long-term clinical management.

The differential diagnosis includes benign endometrial polyps, adenofibroma, adenosarcoma, complex atypical endometrial hyperplasia (CAH), malignant endometrial mixed tumour (MMMT) and EC [25]. In some patients, it is difficult to distinguish APA from cervical polyps when the lesions protrude from the cervix into the vagina. APA occurs in young, nulliparous and premenopausal women, and the sectioned surface is solid, polypoid, firm, rubbery or lobulated, whereas adenofibroma, adenosarcoma, MMMT and EC typically occur in postmenopausal women with large exophytic masses or endogenous infiltrative lesions. According to the literature, EC arising from adenomyosis is associated with significantly younger onset ages and better survival than other cases where adenomyosis coexists with EC. This distinctive behaviour between the two conditions may suggest that when EC arises from the adenomyotic microenvironment, the degenerated stromal cells could have a less aggressive phenotype and could be more susceptible to hormonal influence [26]. Vascular ultrasound is easy to perform and interpret and may improve the detection rate of EC in perimenopausal and postmenopausal women [27]. In contrast to the increased cellularity, cytological atypia and short interlacing fascicles of stroma in APA, typical endometrial polyps and adenomyomas comprise benign endometrial glands, myomatous stroma and a minor component of fibrous tissue. Squamous metaplasia occurs in more than 90% of patients with APA, while it is uncommon in those with other benign lesions, so squamous metaplasia is another useful marker for the differential diagnosis.

Two to 5 years after surgical treatment is the peak time of APA recurrence among patients [28, 29]. To avoid APA relapse, close follow-up should be conducted for 5 years. Hysteroscopy and endometrial biopsy should be performed within 3 to 6 months after treatment. If endometrial abnormalities are not diagnosed during two consecutive examinations and if the patient has fertility requirements, is of reproductive age and does not have any infertility factors, it is recommended that the patient actively attempt natural conception or conception by assisted reproductive technology. Therefore, the patient should be followed up by regular transvaginal ultrasound examinations to monitor changes in the endometrium. Moreover, once the patient develops abnormal uterine bleeding, an abnormal intrauterine echo and other symptoms, she needs to undergo both hysteroscopy and endometrial biopsy to obtain evidence of endometrial abnormalities. On the other hand, if precancerous endometrial lesions or EC are found during two consecutive examinations, the effective treatment plans should be further determined according to age, pathological diagnosis and fertility requirements.

However, there are some limitations in our study. This was a retrospective study. The sample size of patients was small, and conservative treatment was not uniform. A large sample size is required for observation and verification of conservative treatment and follow-up of APA patients.

## Conclusions

For patients with intracavitary lesions > 1 cm, the hysteroscopic four-step diagnosis and treatment strategy and pathological diagnosis are the basis of clinical treatment. More than 30% of APA surface glands have complex structures characterized by branching and budding or other high-risk factors, such as endometrial hyperplasia, which are indications for hysterectomy. For patients who desire to become pregnant or preserve the uterus, hysteroscopy with complete excision of the lesions should be the preferred treatment. The patients should be treated individually and undergo close follow-up, and they should be followed up by regular hysteroscopy and endometrial biopsy.

## Data Availability

The datasets used and analysed during the current study are available from the corresponding author on reasonable request.

## Abbreviations

APA: Atypical polypoid adenomyoma
AUB-O: Abnormal uterine bleeding due to ovulatory dysfunction
BMI: Body mass index
